# The effects of an enrichment training program for youth football attackers

**DOI:** 10.1371/journal.pone.0199008

**Published:** 2018-06-13

**Authors:** Diogo Coutinho, Sara Santos, Bruno Gonçalves, Bruno Travassos, Del P. Wong, Wolfgang Schöllhorn, Jaime Sampaio

**Affiliations:** 1 Department of Sports Sciences, Exercise and Health, University of Trás-os-Montes and Alto Douro, Vila Real, Portugal; 2 CIDESD, Research Center in Sports, Health Sciences and Human Development, Portugal; 3 University Institute of Maia, ISMAI, Maia, Portugal; 4 Department of Sports Sciences, University of Beira Interior, Covilhã, Portugal; 5 Sport Science Research Center, Shandong Sport University, Jinan, China; 6 Institute for Training and Movement Science, Johannes Gutenberg-University, Mainz, Germany; Sao Paulo State University - UNESP, BRAZIL

## Abstract

The aim of this study was to identify the effects of a complementary training program based on differential learning approach in the physical, technical, creative and positioning performance of youth football attackers. Fifteen players were allocated into the control (U15C = 9, age: 13.9±0.5 years; U17C = 6, age: 16.1±0.7 years) and the experimental (U15E = 9, age: 14.2±0.8 years; U17E = 6, age: 15.8±0.5 years) groups. The experimental groups participated in 10-weeks of a complementary training program based on differential learning approach to improve physical literacy and players’ tactical behavior. Variables studied encompassed: motor (vertical jump, speed and repeated change-of direction), technical (pass, dribble and shot), creative (fluency, attempts, versatility) and positioning-related variables (stretch index, spatial exploration index and regularity of the lateral and longitudinal movements). Results revealed that U15E improved both the jump and repeated change-of-direction performance, while the U17E have only improved the jump performance. The U15E showed improvements in all technical variables (small to large effects), and in the fluency and versatility (moderate effects), while the U17 have only improved the successful shots (large effects). From a positional perspective, there was a moderate increase in the stretch index, and decreased longitudinal and lateral regularity (small to moderate effects) in the U15E compared to the U15C. In turn, the U17E revealed a moderate increase of the spatial exploration index and a small decrease in the stretch index. Overall, the results suggest that the complementary training program was effective for the development of the overall performance of the U15E attackers, while more time and/or variability may be needed for older age groups. Nevertheless, the overall higher values found in experimental groups, may suggest that this type of complementary training program improves performance.

## Introduction

Football is a team sport where two opposing teams dynamically compete for space and time to gain advantage over the opponents [1]. Nowadays, performance is being analyzed by describing how players adapt their movement behaviors based on the dynamic configurations of play [1, 2] and changes in the environment [3]. The game has become faster, more intense and compact during the last years [4], which requires from players more abilities to interact with the teammates and opponents to maintain functionality of performance. According to that, training programs and practice tasks designed to improve the players’ overall performance should be focused on promoting adaptability through an increase on variability of goals or contexts of practice [3, 5–7]. Especially, the increasing use of defensive strategies, the concentration of players in the central zones of the pitch and the numerical unbalance that attackers usually face [4, 8], requires players with more unpredictable and creative movement patterns.

In fact, to the attackers is assigned the ultimate responsibility of scoring goals when in possession and need to act as the first element of defense when out of possession [9]. For accomplishing these various type of specific behaviors, the youth attackers cover around 8000 m during the game, with 686 m being above 19.1 km.h^-1^ [10]. This ability to cover distance at high intensities seems to be of high importance in youth attackers [10], once the sprinting ability has been considered a key performance indicator in goal situations [11]. This data highlights the importance of the physical performance of youth attackers positional role. From the technical perspective, during the match the attackers perform an average of 26 to 44 passes (70 to 82% of accuracy), 1 to 2 dribbles, 1 to 2 shots on target and 87 to 90 touches in the ball per match [12, 13]. Furthermore, their proximity to the opponent’s target and defense require them to perform more versatile and creative technical actions so that they may become more unpredictable to their opponents [8, 14, 15]. From the tactical perspective, youth attackers are also recommended to develop more irregular movement patterns [16], which seems to emerge from the continuous attempt of create unpredictable situations to create open space in the offense [14, 15]. Overall, coaches should take into consideration the previous assumptions when designing training tasks and programs aiming to develop the overall youth attackers performance, which can be nurtured with tasks that develop physical literacy, players’ creativity and adaptive behaviors [5, 17].

The physical literacy is characterized by the ability of an individual to behave with confidence in a range of novel and challenging environments, from which he uses the available key information to sustain the emergence of movement behaviors [5, 17, 18]. In the soccer context, the physical literacy consists of combining the fundamental movement skills (i.e., running, sprinting) with fundamental game skills (i.e., knowing when and how to dribble an opponent, how to create space and passing lines) to spatial-temporal conditions of the game context (i.e., angles and distances to opponents, and teammates, distance to the target). Physical literacy encompasses the development of the fundamental movement and game skills, which are determinant most important for the youth attackers, and have been related with a higher increase of the game performance [8, 11, 19], as well as with creative behavior disposition [17]. For that, the training tasks should promote the development of players’ ability to effectively apply cognitive, motor and perceptual skills in dynamic game environments that increases players adaptability. Therefore, the differential learning seems to emerge as a useful approach to promote the previous mentioned contexts.

Differential learning explores increased fluctuations in the players’ movement patterns, requiring from them adaptive mechanisms in the perception-action system by no movement repetition and without corrections during the learning process [5, 20–24]. That is, this approach aims to increase the level of noise, so that the player is challenged to continuously adapt his movement behavior, leading to novel movement configurations 0 For example, different boundary conditions of the body (e.g., both hands behind the head, with one hand in the shoulder, with hands on the hips), ball types (e.g., rugby, tennis, reflex ball), and material (e.g., wearing glasses, without trainers) can be used during a finishing task to increase the adaptive mechanisms that sustain the performance at each shot. Accordingly, differential learning based improvements in technical [6] and motor performance have been earlier reported [6]. Also, an enrichment training program, the Skills4Genius, embraced the differential learning as one of its tenets for a five months period in under-10 years old school-based children [17]. The results revealed improvements in agility and speed as well as in the creative thinking which was assessed through the Torrance Test of Creative Thinking, showing that this training program had influence on the improvement of the players’ general creativity. Also, children showed more versatile actions as well as higher movement coordination with the teammates. More recently, a study compared the impact of a 5-month training intervention based on differential learning embedded in small-sided games with a intervention based on a more traditional approach in youth soccer players (under-13 and under-15) [23]. The results shown improvements in players technical actions, in their creative predisposition, as well as more regular tactical performances, while decreasing the number of unsuccessful actions [23]. Overall, the aforementioned studies showed that differential learning was effective in developing physical, technical, creative and tactical performance of school-based children and youth soccer players. Taking into consideration the positive benefits of this training program in youth, as well as the noted improvements, it is possible that training programs sustained in differential learning might also be efficient to develop the specific playing positions performance, such as attackers.

Furthermore, attackers are a playing position in which different and unexpected movement behaviors are desired to break the symmetry with the defenders [14, 15]. Accordingly, the differential learning seems to boost the creative potential of players [5, 23], however, no study has inspected the potential effects of this approach to improve attackers creative behavior. Additionally, it is later in the players’ development process that players seem to acquire a better understanding of their positional role. In fact, the age of 16 years old has been considered a critical phase in the development process of players [25], and thus it is important to analyze the effects of enrichment training programs that aims to improve youth players performance. Therefore, this study aimed to analyze the effects of a training program sustained in physical literacy and differential learning in the physical, technical, creative and tactical performance of under-15 (U15) and under-17 (U17) youth football attackers.

## Methods

### Participants

The participants included 86 young male Portuguese football players from U15 and U17 age groups. All players belong to two different youth clubs competing in a Portuguese regional level (2014/2015 season). The criterion that was used to include the players in this study was their playing position (attackers), therefore, from the 86 players, only 30 (n = 18 for the U15, and n = 12 for the U17) were considered for data analysis (Table 1). Then, the players were allocated into respective control and experimental groups according to their club, that is, the U15 and the U17 players from one club were considered as experimental, while the U15 and U17 from the other club were considered as control. In this sense, both U15 control group (U15C) and U15 experimental group (U15E) consisted of 9 attackers each. In turn, both the U17 control group (U17C) and the U17 experimental group (U17E) were composed of 6 attackers.

**Table 1:** Characteristics of experimental and control groups.

	U15C		U15E		U17C		U17E	
	Pre-Test	Post-Test	Pre-Test	Post-Test	Pre-Test	Post-Test	Pre-Test	Post-Test
**N**	9		9		6		6	
**Age** (years)	13.9±0.5		14.2±0.8		16.1±0.7		170.6±5.8	
**Height** (cm)	167.0±9.0	169.2±11.4	161.2±5.5	162.9±5.4	174.3±5.8	177.3±2.2	170.6±5.8	172.3±5.9
**Mass** (kg)	54.2±11.1	55.7±8.2	50.7±10.3	51.4±10.4	66.9±5.2	65.1±7.2	64.9±6.8	64.9±6.3
**Body Mass Index** (kg/m^2^)	19.2±2.3	19.3±2.4	19.4±2.8	19.2±2.9	21.7±1.7	21.8±1.8	20.7±1.8	21.9±2.6
**Playing Experience** (years)	6.1±3.1		6.4±3.2		8.0±2.1		6.4±3.2	

U15C: under-15 control group; U15E: under-15 experimental group; U17C: under-17 control group; U17E: under-17 experimental group; N: number; cm: centimeters; kg: kilograms; m: meters.

All groups followed the typical football training sessions 3 times a week, with each session lasting ≈ 90 minutes. Moreover, the groups participated in a 70 and 80-minute official game once a week, for the U15 and U17 respectively. In the U15, the training sessions were focused on the development of elementary technical and tactical skills with an overall training time of ~86 minutes (U15C training duration = 84.3±8.5 minute; U15E training duration = 87.9±4.5 minute). Various skills activities (e.g., ball mastery, dribbling, passing and receiving drills) were performed for 30 minutes in all sessions of the week. After this period, the coaches dedicated 30 minutes to develop players technical and tactical skills based on a sectorial level, with the aim of improving elementary principles of offense and defense. The training session ended with 30 minutes of game based situations (e.g., from 5vs5 to 7vs7 small-sided games) in the first and last session of the week, and with 11vs11 simulated game in the middle session. The U17 standard training sessions were composed by 20–30 minutes of technical and physical skills (e.g. sprint and 1v1), 40–50 minutes of team tactical organization, and finished with 20 minutes of small-sided games or continuous play (11v11 a side), with and overall session time of ~89 min (U17C training duration = 89.2±2.5 minute, U17E training duration = 87.9±3.4 minute). An informed consent was provided to the coaches, players, and their parents, as well as by the club, before the study began. All participants were notified that they could withdraw from the study at any time. The study protocol followed the guidelines and was approved by the Local Ethics Committee and conformed to the recommendations of the Declaration of Helsinki.

### Testing procedures

**Fig 1:**
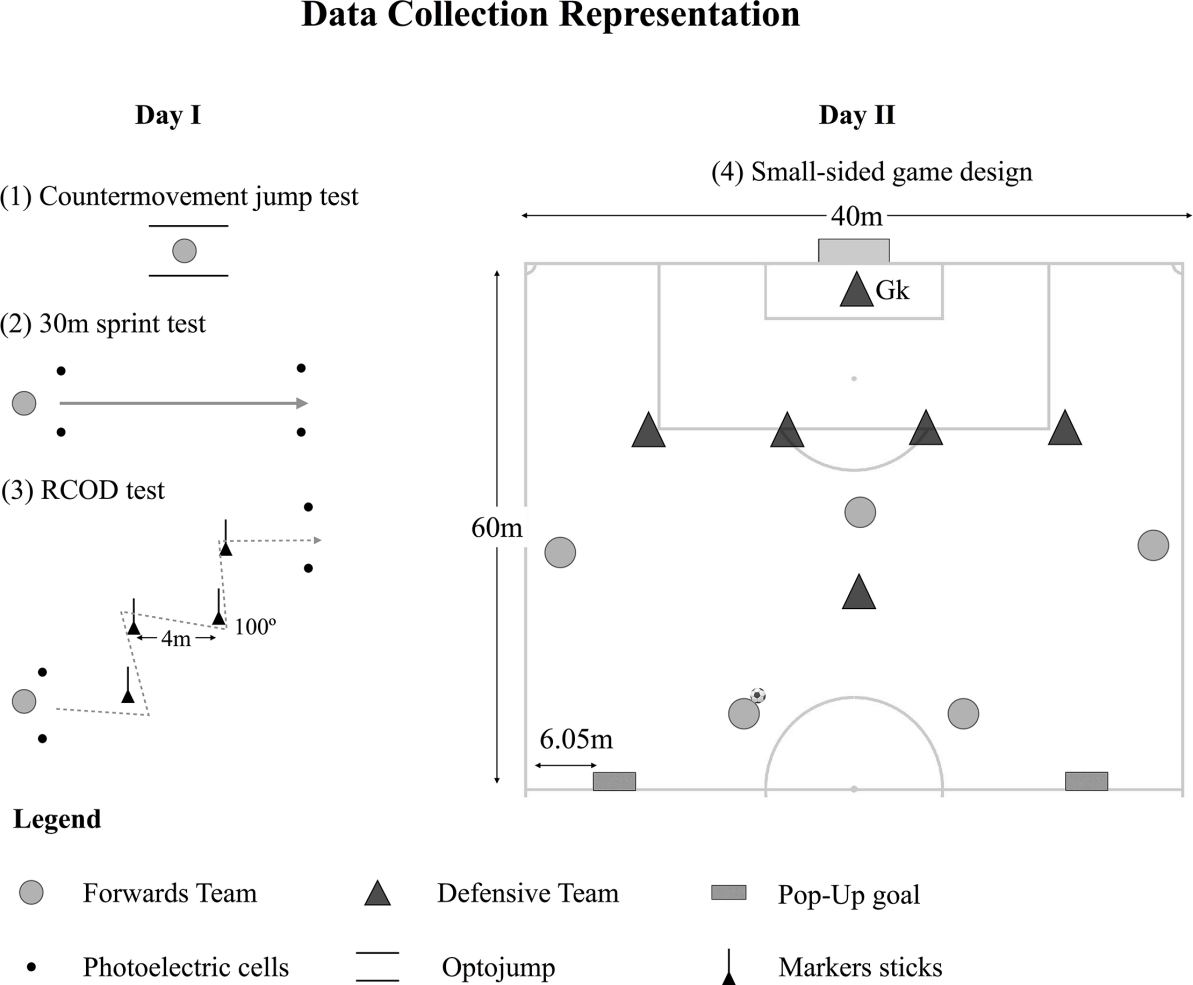
Schematic representation of the data collection tests.

Anthropometric measurements took place before the pre-test session. One week before the pre-test measurement, participants were familiarized with the tests. Before each of the testing sessions, there was a standardized 15-min warm-up based on running and ball possession games without goals (5-a-side). All participants were tested before (pre-test) and after (post-test) of a 10-week experimental period. The pre-post-test measurements were performed in two sessions each. The first session was based on the physical performance measurements, where each player performed the counter-movement jump, a 30m sprint and the repeated change-of-direction (RCOD) tests. The second testing session was used to evaluate the players performance in a game-based situation, and therefore it was performed a 5vs5+Gk small-sided game (SSG) on a 60×40m (length × width) pitch (see Fig 1). In each SSG was one team with the aim to attack and score the regular goal, which was composed by two midfielders and three attackers, while the opposing team with possession has the aim of playing out from the back and score in two small goals (intended to represent the two midfielders of the team), was composed by four defenders and one defensive midfielder. This SSG format was applied to be representative of the game demands on the final third of the pitch, that is, one team seeking to create shooting opportunities with ball possession and put pressure on the opponents in case of being without ball possession. In turn, the opposing team had the aim of progress in the field and defend the regular target.

All SSGs lasted for a total of 24 minutes consisting of three bouts of 6 minutes interspersed with a 3-min passive recovery period. The head coach divided the players into balanced teams, and the players performed each SSG according to their usual playing position role. All SSGs were performed as much as possible with the official game rules, with the one exception of the goal, that independently of the team that scored, it was replaced in the midfield zone by the attackers team to increase the amount of time in their possession. Several balls were placed around the field to ensure it replacement as fast as possible. The coach intervention was minimized and therefore, no feedback was allowed during the game. No coach feedback or encouragement was allowed during the conditions. All the test sessions were performed at the same time period of the day (from 6.30 p.m. to 8.30 p.m.) under similar environmental conditions.

### Training intervention

The study was conducted in the middle of the competitive season and the training program lasted for 10 weeks with 2 sessions per week in non-consecutive days with a duration of 25 minutes each (for a total of 20 sessions). All the sessions were performed in the beginning of the training session, while the other members of the team performed the regular football training conducted by the coach. Afterwards, they joined the rest of the group to continue the training session (for the next 65 minutes). Each training program intervention consisted of the following components: 10 minutes of physical literacy combined with differential learning approach exercises and 15 minutes of SSGs with additional differential learning. All the training sessions started with a 10 minutes period in which the fundamental movement (agility, speed, coordination and plyometric training) and the game skills (dribbling, passing and shooting) were combined in a circuit (see Table 2) and embodied in a differential learning environment. For example, the players had to perform a specific ladder drill exercise, followed by a change-of-direction task and ended in 1v1 situations towards the goal with additional differential learning fluctuations (e.g. dribbling with one arm raised up). Moreover, the tasks and conditions were always changing to provide an unpredictable enriched environment that allowed the player development [17]. For instance, the players faced different circuits, number of oppositions and teammates, attacked from different distances and angles to the target.

**Table 2:** Description of the general training program tenets.

	Physical Literacy	Differential Learning
	Combination of the fundamental movement skills with the fundamental game skills in a wide range of environments;	Small-sided games underpinned in unpredictable and dynamic environments that constantly required adaptation from the players.
Type of Tasks Variation	In this approach, it was combined two fundamental movement skills (e.g. coordination followed by agility) with the fundamental game skills (e.g. 1v1).	• Changing the number (1 to 4) and type of targets (e.g. 11-a-side, pop-up goals, crossing lines with the ball under control);
	• Fundamental movement skills (coordination, plyometric, speed and agility;	• Changing the pitch size (small, medium and large pitches);
	• Fundamental game skills (dribbling, passing, receiving and shooting);	• Changing the pitch shape (e.g. circle, square, diamond);
	Moreover, the tasks were performed in a wide range of contexts:	• Limiting the pitch space (e.g. only allowed play in the corridors; not allowed to play in the central midfield zone);
	• Formats (from 1v1 to 3v3);	• Changing the number of players (from 1v1 to 6v6, superiority and inferiority);
	Balance (inferiority, equal and superiority);	• Modifying the type of ball (e.g. reflex ball, rugby, basketball, tennis);
	Distance (close, medium and large distance to target);	• Finishing with body manipulations (e.g. hands in the back, arm raised);
	• Angle (left, centre and right in relation to the target)	• Performing the task with one eye covered (right and left);
	• Space (small, medium, large)	• Adding obstacles to the pitch (e.g. elastics, ropes, sticks, markers).
	Changing the number (1 to 4) and type of targets (e.g. 11-a-side, pop-up goals, crossing lines with the ball under control);	

Regarding the 15 minutes of SSGs based on differential learning, these game-based scenarios were randomly manipulated (i.e., with continuous changes on practice task constraints) to continuously promote players adjustments and improvisation of new movement patterns [6, 22, 23, 24]. For example, the players performed SSGs added with constantly changing specific body movements (e.g. arms raised up, carrying a ball in the hands, for further details see Table 2). Therefore, all the program training tasks within the program were grounded on movement variability and unpredictability, to increase the attackers’ ability to adapt to the ever-changing environment. All the tasks were also designed to develop players individual and collective positional principles related to their specific positional role such as: a) attacking the goal, b) feinting, c) taking the ball to the goal, d) creating space and time for teammates; e) achieving advantage; f) increasing the effective playing space; g) and attacking together [5, 26]. All the training sessions were fully supervised by an experienced football coach (with 7 years of experience in youth coaching).

### Data collection

#### Vertical jump tests

A *counter-movement jump* (CMJ) was utilized to measure the vertical jump height. The jumping height was measured with a portable optical timing system (Optojum-Next® Microgate, Bolzano, Italy). During the CMJ test the participants were instructed to perform the jump with hands on the hips to eliminate the arm-swing effects [27]. Two jumps for each test were performed with 1-min of recovery between jump, and the best result was considered for further analysis.

#### 30m sprint test

Sprint performance was assessed with two 30m maximum sprints. The time was measured using two pairs of photoelectric cells (Optojump, Microgate, Bolzano, Italy), positioned at 0 and 30m and at a height of 1m. The participants started the sprint from an upright standing position with the front foot placed at 10 cm before the first timing gate. Two repetitions were performed with 3-min of recovery between trials, and only the fastest sprint was considered.

#### Repeated change-of-direction test

The RCOD comprised 6 sprints of 20m with 25 seconds of active recovery. The test consisted of four 100° change of directions at every 4m [28]. The sprint time for each sprint was determined with two pairs of photoelectric cells (Optojump, Microgate, Bolzano, Italy), placed at the starting and finishing lines (0 and 20m), 1m above the ground. The subjects started the sprints from an upright standing position and with the front foot 0.5m before the first pair of photocells [28]. After each sprint, the participant slowly jogged back to the starting line.

#### Creative behavior and technical game performance

The SGG were recorded using a digital video camera, Sony NV-GS230, from a fixed position and at a 2m height and aligned in the central zone of the pitch. Afterwards, the video files were downloaded to a computer and a notational analysis was performed using the LongoMatch software (Longomatch, version 1.3.7., Fluendo). The following creative components were assessed based on the individual technical actions recorded: a) fluency, considered as the ability to execute as many successful actions as possible; b) attempts, or any effort to perform different actions, but non-successful; and c) versatility, recognized as ability to produce a diversity of actions, different from the standard movements, with success [5, 17]. Accordingly, the successful actions were considered as fluency, while the unsuccessful different actions were considered as attempts, and the versatility comprised the successful different movement patterns (check for referencing Santos et al. [17], [23]).

Also, the following individual team performance variables were registered: successful passes, dribbles, and goal shots, as well as the number of unsuccessful passes, dribbles and goal shots were collected [13]. The previous variables were selected due to their relevance on the attackers’ offensive performance. The data were gathered by experienced performance analyst, and the reliability of the data were inspected by retesting 17.5% of the sample. The intra-class correlation was deemed as high (>0.88) [29].

#### Positioning behavior

Positional data during SSGs were gathered using 5 Hz Global Positioning System (GPS) units (SPI-PRO, GPSports, Canberra, ACT, Australia). The players’ latitude and longitude coordinates obtained through the GPS units were exported and processed using appropriate routines in Matlab® (MathWorks, Inc., Massachusetts, USA) (check for referencing Folgado et al. [1]). The dynamic positional data from the players were used to determine the attackers’ stretch index per minute [30], the spatial exploration index (SEI) [2] and the longitudinal and lateral regularity in the players’ movement patterns [31]. These two last variables were assessed by applying the Approximate Entropy processing technique (ApEn), which allows to understand if the players’ movement displacements in the lateral and longitudinal directions exhibit more regular or irregular patterns. The imputed values were 2 for the vector length (m) and 0.2*standard deviation for the tolerance (r) [32]. This variable value ranges from 0 to 2, and values closer to 0 means that players behave more regular and are likely to repeat the same movement patterns on the pitch [31].

### Statistical analysis

The comparisons between the groups (CG vs. EG, for each age group) were analyzed with a specific spreadsheet for pre-post parallel groups trial [33, 34]. For the motor and positional related variables, the effects were estimated in percent units through log-transformation (to reduce the non-uniformity of error) and uncertainty in the estimate was expressed as 90% confidence limits, while both the creative and technical variables were presented as absolute raw values. Smallest worthwhile differences were measured using the standardized units multiplied by 0.2. Uncertainty in the true effects of the conditions was assessed based on non-clinical magnitude-based inferences. Probabilities were reported using the following scale: >5%, unclear; 25 to 75%, possible; 75% to 95%, likely; 95% to 99%, very likely; >99%, most likely [33]. Standardized (Cohen) mean differences, and respective 90% confidence intervals were also computed as magnitude of observed effects, and, thresholds were: <0.2, trivial; 0.6, small; 1.20, moderate; 2.0, large; and >2.0, very large [33].

## Results

### Physical performance

**Fig 2:**
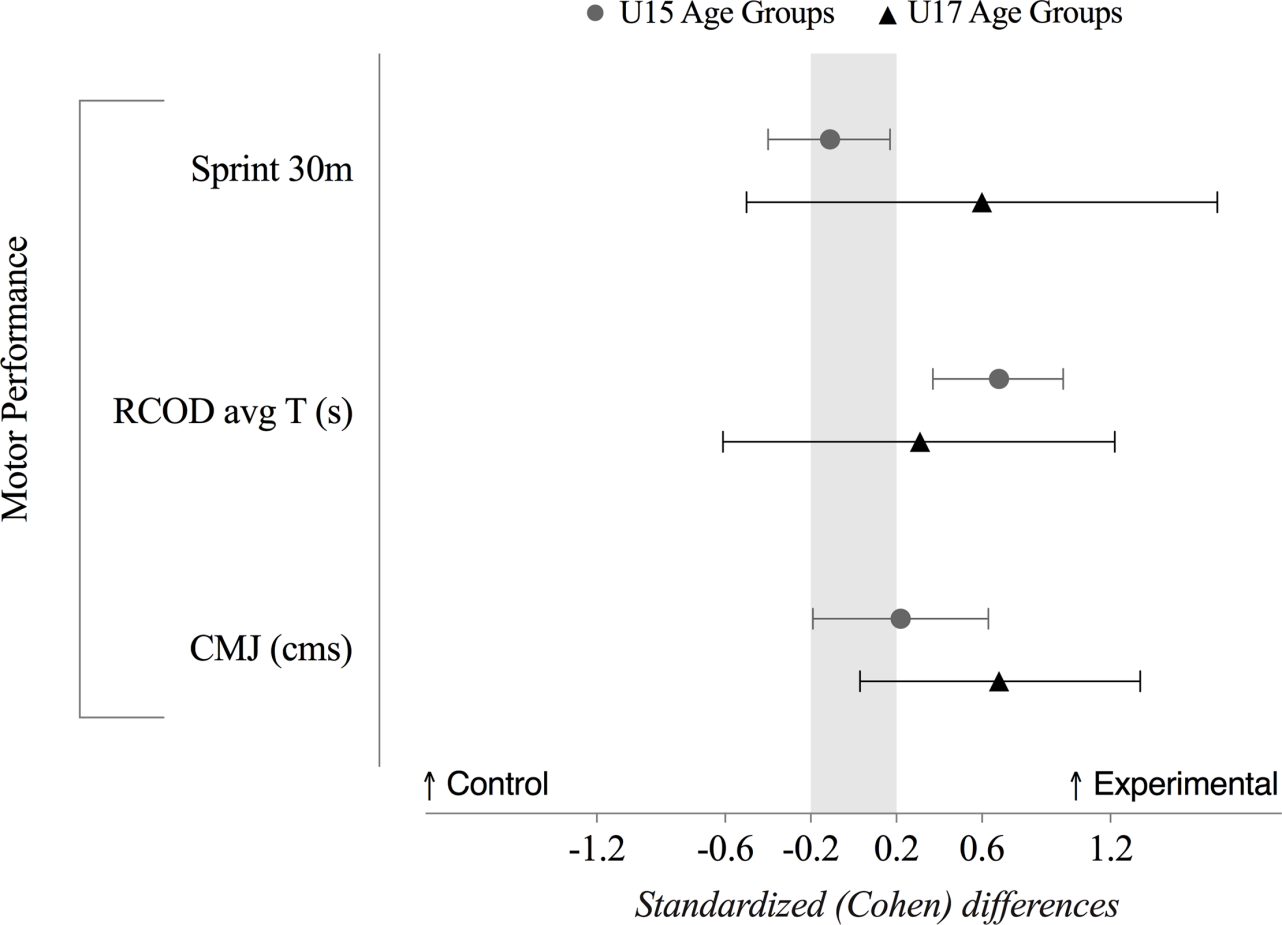
Standardized (Cohen’s *d*) differences of the motor performance between the control and experimental groups (grey ⦁ dots represent U15 age groups, while the ▲ black triangles represent the U17 age groups). Error bars indicate uncertainty in the true mean changes with 90% confidence intervals. Light grey zone reflects trivial values. Values towards the right means higher values for the experimental group, while higher values towards the left means higher values for the control group. RCOD: repeated change-of-direction; CMJ: counter-movement jump.

The effects of the training program on attackers’ physical performance in the U15 and U17 age groups are presented in Fig 2, Table 3, Table 4, Table 5 and Table 6. Overall, the results revealed that both control (CG) and experimental (EG) groups in the U15 improved their performance. However, the improvements were higher in the U15E. In this sense, the U15E presented a moderate improvement in the average time needed to perform the repeated change of direction (RCOD) (change in means, %; ±90% confidence limits: possibly, -4.1%; ±1.8%) compared to the U15C. The training program also showed moderate improvement in the height of CMJ in the U17E (likely, 8.4%; ±8.4%) compared to the U17C.

**Table 3:** Descriptive statistic for U15 group comparisons in physical and technical performance, creativity components and positional variables.

Variables	U15 Control Group			U15 Experimental Group		
	Pre-Test	Post-Test	Percentage Variations from Pre to Post-Test	Pre-Test	Post-Test	Percentage Variations from Pre to Post-Test
	(Mean±SD)	(Mean±SD)	(Mean±SD)	(Mean±SD)	(Mean±SD)	(Mean±SD)
**Physical Performance**						
Sprint 30m	4.85±0.47	4.62±0.34	3.5±3.1	4.94±0.30	4.82±0.32	-2.5±2.9
RCOD avg T(s)	6.55±0.33	6.41±0.36	-1.9±2.3	6.74±0.42	6.34±0.38	-5.9±2.2
CMJ (cm)	32.38±4.85	35.01±5.18	7.0±9.6	29.39±5.34	32.53±4.27	11.4±9.2
**Technical Variables**						
Successful dribbles (n)	0.43±0.77	0.17±0,38	-0.4±1.4	0.22±0.42	0.41±0.69	0.3±1.2
Successful shots (n)	0.47±0.68	0.23±0.50	-0.4±1.2	0.19±048	0.70±0.67	0.8±1.4
Goals (n)	0.30±0.47	0.10±0.31	-0.5±1.3	0.11±0.32	0.11±0.32	0.2±1.1
**Creative Components**						
Fluency (n)	3.5±2.35	3.17±1.86	-0.2±1.2	3.00±1.78	4.26±2.07	0.6±1.4
Attempts (n)	0.23±0.50	0.17±038	-0.1±1.4	0.22±0.42	0.26±0.53	0.1±0.4
Versatility (n)	0.40±0.72	0.23±0.43	-0.3±1.3	0.11±0.42	0.41±0.57	0.5±1.1
**Positional Variables**						
SEI (m)	10.99±1.48	11.27±1.43	1.6±18.6	10.47±1.84	10.52±1.17	1.5±23.7
Stretch Index (m)	10.01±1.35	9.38±1.37	-5.3±21.4	10.37±1.20	10.63±1.15	2.5±17.8
Lateral Regularity (ApEn)	0.11±0.02	0.09±0.02	-10.2±24.7	0.11±0.02	0.11±0.2	-0.2±25.8
Longitudinal Regularity (ApEn)	0.13±0.04	0.11±0.02	-16.6±44.9	0.13±0.03	0.14±0.13	5.2±32.8

**Note**: RCOD avg T = repeated change-of-direction task average time; CMJ = counter-movement jump; SEI = spatial exploration index; ApEn = approximate entropy.

**Table 4:** Inferential statistic for U15 group comparisons in physical and technical performance, creativity components and positional variables.

Under-15			
(Control Group vs Experimental Group)			
Variables	Difference in means: %; ±90% CL	Chances, % Decrease / Trivial / Increase	Practical Inferences
**Physical Performance**			
Sprint 30m	1.0; ±2.5	34/61/4	Possibly increase
RCOD avg T(s)	-4.1; ±1.8	0/1/99	Very likely increase
CMJ (cm)	4.1; ±8.0	6/38/56	Unclear
**Technical Variables**			
Successful dribbles (n)	0.5; ±0.4	1/7/93	Likely increase
Successful shots (n)	0.8; ±0.4	0/0/100	Most likely increase
Goals (n)	0.2; ±0.2	3/17/80	Likely increase
**Creative Components**			
Fluency (n)	1.6; ±1.2	0/5/94	Likely increase
Attempts (n)	0.1; ±0.3	13/34/53	Unclear
Versatility (n)	0.5; ±0.3	0/3/97	Very likely increase
**Positional Variables**			
SEI (m)	-0.1; ±9.1	28/46/26	Unclear
Stretch Index (m)	8.2; ±6.2	0/6/94	Likely increase
Lateral Regularity (ApEn)	11.2; ±11.9	1/14/85	Likely increase
Longitudinal Regularity (ApEn)	26.1; ±20.2	0/3/97	Very likely increase

**Note**: RCOD avg T = repeated change-of-direction task average time; CMJ = counter-movement jump; SEI = spatial exploration index; ApEn = approximate entropy. Change in means were presented as %; ±90% CL for the physical and positional variables based on log-transformed data, whereas for the technical variables and creative components were used as absolute values (with ±90% CL).

**Table 5:** Descriptive statistic for U17 group comparisons in physical and technical performance, creativity components and positional variables.

Variables	U17 Control Group			U17 Experimental Group		
	Pre-Test	Post-Test	Percentage Variation from Pre to Post-Test	Pre-Test	Post-Test	Percentage Variations from Pre to Post-Test
	(Mean±SD)	(Mean±SD)	(Mean±SD)	(Mean±SD)	(Mean±SD)	(Mean±SD)
**Physical Performance**						
Sprint 30m	4.33±0.07	4.23±0.23	-2.4±4.5	4.94±0.30	4.82±0.32	-4.7±2.1
RCOD avg T(s)	6.25±0.36	6.12±0.29	-3.6±4.5	6.74±0.42	6.34±0.38	5.3±4.2
CMJ (cms)	40.98±1.69	38.58±2.98	-6.0±5.3	29.39±5.34	32.53±4.27	1.9±8.8
**Technical Variables**						
Successful dribbles (n)	0.17±0.51	0.28±0,57	0.2±1.0	0.22±0.42	0.41±0.69	0.2±1.6
Successful shots (n)	0.39±0.61	0.06±0.24	-0.7±1.1	0.19±048	0.70±0.67	-0.1±1.3
Goals (n)	0.17±0.38	0.17±0.38	0.0±1.3	0.11±0.32	0.11±0.32	-0.1±0.6
**Creative Components**						
Fluency (n)	3.78±2.07	3.89±2.54	0.1±1.3	3.00±1.78	4.26±2.07	-0.2±1.2
Attempts (n)	0.11±0.17	0.17±0.38	0.2±1.4	0.22±0.42	0.26±0.53	-0.4±1.3
Versatility (n)	0.17±0.51	0.28±0.75	0.2±1.8	0.11±0.42	0.41±0.57	0.3±1.5
**Positional Variables**						
SEI (m)	9.64±1.20	9.96±1.80	1.3±14.2	10.47±1.84	10.52±1.17	16.9±17.1
Stretch Index (m)	9.15±1–00	8.95±1.14	-2.4±16.4	10.37±1.20	10.63±1.15	-8.3±20.2
Lateral Regularity (ApEn)	0.11±0.02	0.11±0.02	-5.4±17.2	0.11±0.02	0.11±0.2	-11.0±26.7
Longitudinal Regularity (ApEn)	0.16±0.04	0.14±0.05	-12.8±25.6	0.13±0.03	0.14±0.13	-20.7±26.7

**Note**: RCOD avg T = repeated change-of-direction task average time; CMJ = counter-movement jump; SEI = spatial exploration index; ApEn = approximate entropy.

**Table 6:** Inferential for U17 group comparisons in physical and technical performance, creativity components and positional variables.

Under-17			
(Control Group vs Experimental Group)			
Variables	Difference in means: %; ±90% CL	Chances, % Decrease / Trivial / Increase	Practical Inferences
**Physical Performance**			
Sprint 30m	-2.3; ±4.2	77/12/11	Unclear
RCOD avg T(s)	-1.6; ±4.8	61/21/18	Unclear
CMJ (cms)	8.4; ±8.4	2/7/91	Likely increase
**Technical Variables**			
Successful dribbles (n)	0.1; ±0.6	28/31/41	Unclear
Successful shots (n)	0.4; ±0.4	2/12/86	Likely increase
Goals (n)	-0.1; ±0.2	44/39/17	Unclear
**Creative Components**			
Fluency (n)	-0.6; ±1.7	54/30/16	Unclear
Attempts (n)	-0.2; ±0.3	79/15/5	Unclear
Versatility (n)	0.1; ±0.5	30/26/44	Unclear
**Positional Variables**			
SEI (m)	15.4; ±9.7	0/1/99	Very likely increase
Stretch Index (m)	-6.1; ±6.3	84/14/2	Likely decrease
Lateral Regularity (ApEn)	-6.0; ±10.9	66/25/9	Unclear
Longitudinal Regularity (ApEn)	-9.0; like±14.4	67/27/6	Unclear

**Note**: RCOD avg T = repeated change-of-direction task average time; CMJ = counter-movement jump; SEI = spatial exploration index; ApEn = approximate entropy. Change in means were presented as %; ±90% CL for the physical and positional variables based on log-transformed data, whereas for the technical variables and creative components were used as absolute values (with ±90% CL).

### Technical performance

**Fig 3:**
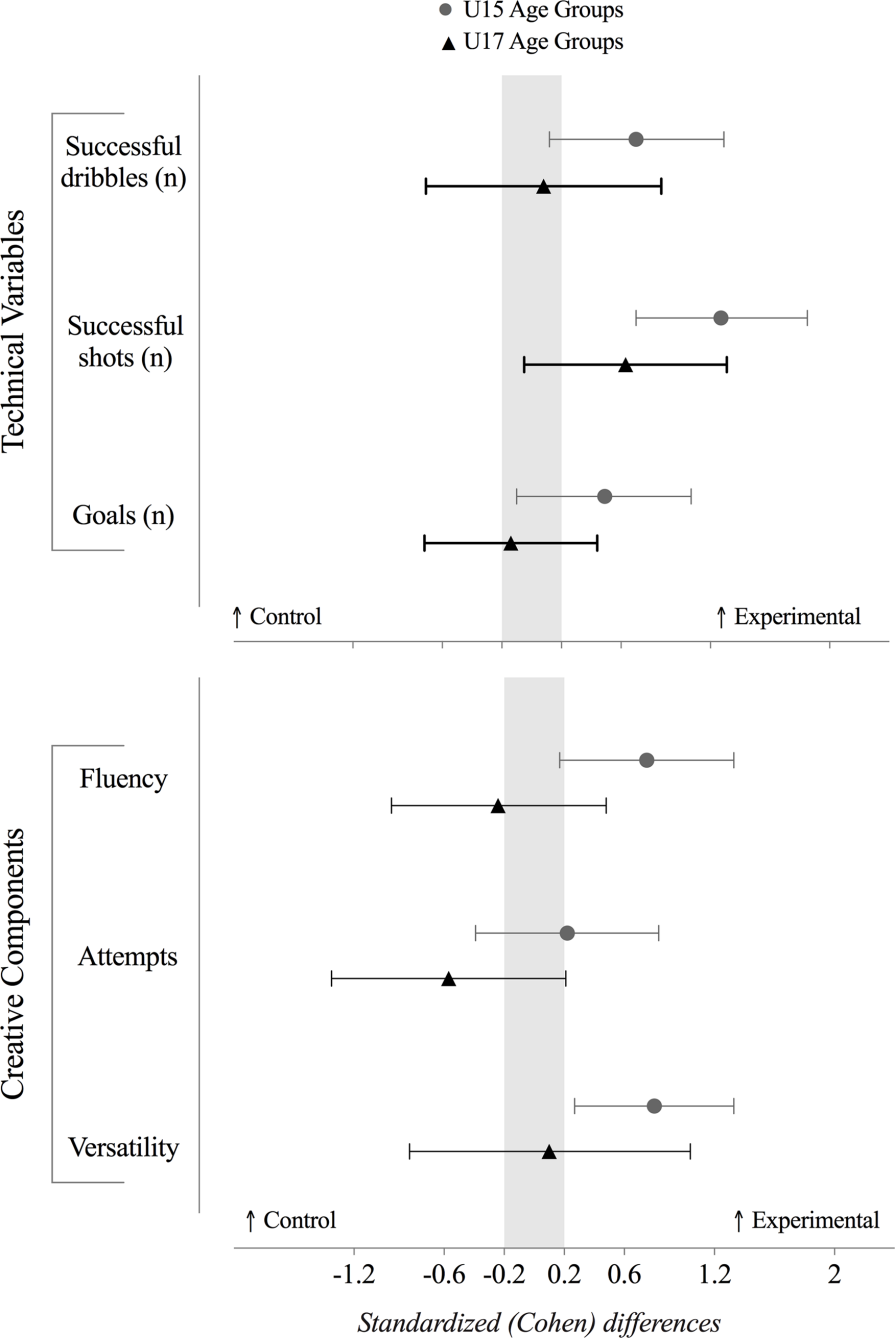
Standardized (Cohen’s *d*) differences of the technical and creative components performance between the control and experimental groups (grey ⦁ dots represent U15 age groups, while the ▲ black triangles represent the U17 age groups). Error bars indicate uncertainty in the true mean changes with 90% confidence intervals. Light grey zone reflects trivial values. Values towards the right means higher values for the experimental group, while higher values towards the left means higher values for the control group. N: number of actions.

The effects of the training program on attackers’ technical performance in the U15 and U17 age groups were presented in Fig 3, Table 3, Table 4, Table 5 and Table 6. The results from both group comparisons show an improvement of the scores for the technical performance of the players, mainly in the U15E. While all technical actions from U15C impaired, for the U15E was observed an improvement. A moderate improvement was depicted in successful dribbles (change in means, absolute value; ±90% confidence limits: likely, 0.5; ±0.4), a large improvement in the successful shots (most likely, 0.8; ±0.4) and a small improvement in the number of goals scored (likely, 0.2; ±0.2) for the U15E. Unclear effects was observed between for successful dribbles and goals. However, the training interventions lead to a moderate improvement of the number of successful shots (likely, 0.4; ±0.4).

### Creative components

The effects of the training program on attackers’ creative components in the U15 and U17 groups are presented in Fig 3, Table 3, Table 4, Table 5 and Table 6. The results showed a moderate improvement of fluency (likely, 1.6; ±1.2) and versatility (very likely, 0.5; ±0.3) for the U15E group. In turn, unclear effects were found in all creative components for the U17 group.

### Positional variables

**Fig 4:**
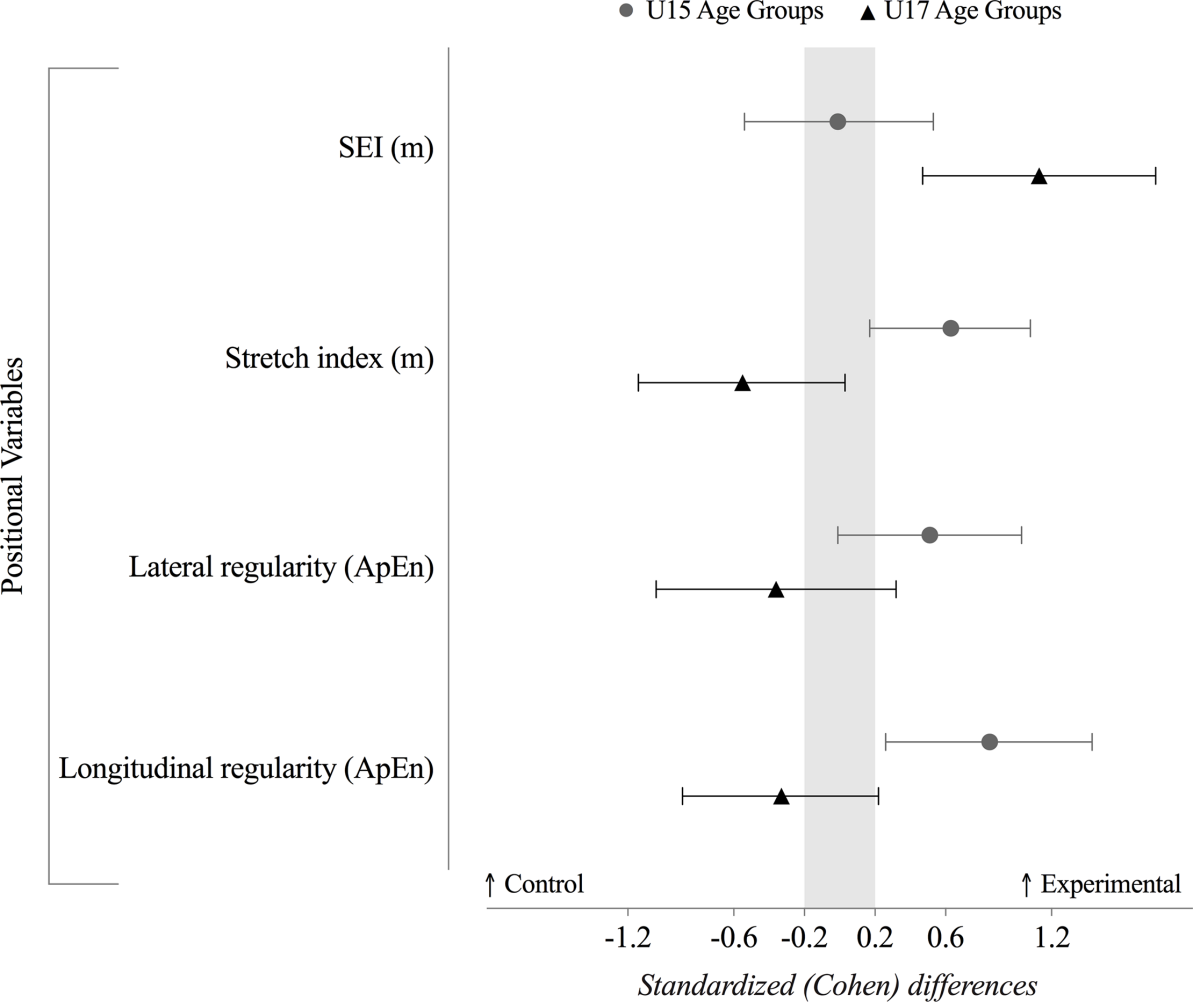
Standardized (Cohen’s *d*) differences of positional variables between the control and experimental groups (grey ⦁ dots represent U15 age groups, while the ▲ black triangles represent the U17 age groups). Error bars indicate uncertainty in the true mean changes with 90% confidence intervals. Light grey zone reflects trivial values. Values towards the right means higher values for the experimental group, while higher values towards the left means higher values for the control group. SEI: spatial exploration index; ApEn: approximate entropy.

The effects of the training interventions on attackers’ positional variables in the U15 and U17 age groups can be seen in detail in Fig 4, Table 3, Table 4, Table 5 and Table 6. The scores of the U15E group showed a moderate increase in the stretch index (likely, 8.2%; ±6.2%) and in longitudinal regularity (very likely, 26.1%; ±20.2%, meaning that the players become more irregular in their movements) as well as a small increase in the lateral regularity (likely, 11.2%; ±11.9%). Regarding to the U17 age group, the U17E revealed a moderate increase in the SEI (very likely, 15.4%; ±9.7%). However, the U17E also revealed a small decrease in the team stretch index (likely, -6.1; ±6.3%), showing that the U17C have presented a higher dispersion in the pitch comparatively to the U17E.

## Discussion

The aim of this study was to analyze the effects of a training program sustained in physical literacy and differential learning in the physical, technical, creative and tactical performance of under-15 (U15) and under-17 (U17) youth football attacker players. Overall, the training program was more effective to the U15, since overall better physical, technical and creative performances were found after the complementary training program. Although the U17 have not shown the same effects of the U15, important results in physical and technical performances were also found. Additionally, different positional movement behaviors were found after the training program in both age groups. Therefore, these results suggest that the training program based on differential training and physical literacy was effective to the overall development of attackers performance, mostly in the U15 age group.

From the physical performance perspective, it was shown higher improvements in the RCOD average time for the U15E, than the U15C. The RCOD requires agility and inter-limb coordination [35], therefore it is possible that the decrease in the time found in the U15E resulted from the increased individual coordination and increased anticipation of changes in directions induced by the training program. In addition, the RCOD seems to be age dependent [35], and therefore it is possible that higher levels of inter-limb coordination can be found in the U17 age group, which could help to explain the unclear trends in this age group [35].

The results from the CMJ revealed higher values after the training program in both experimental groups, which are in line with the reports investigating the effects of short- term (from 8 to 12 weeks) training programs based on plyometric and speed activities in U15 [36] and U17 [37]. In addition, there was a decrease in the CMJ in the U17C, and thus training programs with the goal to improve physical literacy may be important to avoid decreases in jumping performance in this age group specifically and in speed abilities in general.

From the technical perspective, the U15E increased the number of successful dribbles, shots and goals, which resulted from the assumptions underpinned in the physical literacy and differential learning approaches. That is, improvements on physical literacy encompasses the perceptual and decision-making capacities of attackers [5], allowing them to better adjust when and how to act. Moreover, the core idea of the differential learning is to increase the fluctuations [5, 6, 20, 22], which may have improved the U15E attackers’ ability to adjust their performance, once the player has the opportunity to explore and continuously adapt their movement patterns [17, 20]. In contrast, lower effects were found in the U17E, nevertheless, the training program was effective to increase the number of successful shots in this age group. Accordingly, the unpredictable and dynamic situations provided by the differential learning and physical literacy approaches, seem to enhance the players’ ability to find successful performances under constantly changing boundary conditions [6, 20].

The mastery of both fundamental technical and tactical skills has been linked with higher in-game performances [8, 11, 19], and a requirement for the creative behavior [17, 23]. Thus, the beneficial effects of the training program in the creative components in the U15E may be linked with previously mentioned improved abilities. For instance, the results showed improvements in the U15E fluency and versatility. This is in line with the available literature that showed that the variability and adaptability induced by differential learning leads to the higher probability of emergence of creative components on performance [5, 17, 23]. On the contrary, no effects were found in the U17E age group. As previously noted, a decrease in the creative behavior occurs with an increase in the age of players, possibly as a result of an increase in convergent thinking [5, 38], and focus in team tactical behavior organization [39]. Another possible contribution may be related with an earlier lack of fluctuations in the training during previous stages, decreasing the players ability to explore and improvise. In fact, it has been demonstrated that players’ earlier sport experiences have an important role on their predisposition to be adaptive [19].

The results from the positioning behavior revealed an increase in the team dispersion in the U15E. The differential learning approach exposes players to unfamiliar scenarios, leading to the emergence of new functional adaptive movement behaviors [6, 7, 20, 23]. Considering that players are encouraged to find their momentary optimal solution for a given task [20, 32, 40], it is likely that players become more versatile and skillful in using the available space after the training program. Also, higher unpredictability in the longitudinal and lateral movements were observed in the U15E, which has been linked with a desirable patterns required in attackers [16]. That is, defenders will find more difficult to stop an attacker when his movement behavior is more unpredictable, and consequently, harder to anticipate [14]. The training program has also revealed effects on the U17E, which showed an increase of the spatial exploration index. Previously, it was suggested that attackers should develop irregular, creative and atypical/unusual movement behaviors to change the interpersonal relation with the defenders [14]. Such results may reveal that U17E increased the area to modify the interpersonal coordination with the defender. In addition, and considering that the U17E showed a more predictable positioning, lower versatile profile in technical actions and lower team dispersion found during the SSGs, increasing the area explored may have emerge as solution to disrupt the balance with the defender.

While the current report provides clear evidence for positive effects of the training program on the physical, technical, creative and tactical performances, some limitations should be acknowledged. Firstly, in this study forward players were considered all the players that play in the front positions, such as the wingers. Further studies should apply this type of training programs for different players’ positions in order to identify possible differences on the evolution of players with different profiles of play. Finally, the training program showed small effects in the U17E team. It is possible that older age groups, with whom more structured training practices are usually adopted, may require training programs with higher duration or even more fluctuations.

## Conclusions

Overall, training programs based on differential learning and physical literacy seem to have the potential to boost attackers in-game performance. The results from this study proved this assumption, as there were improvements in the physical, technical, creative and tactical performances, mainly in the U15 age group. As so, coaches could use training programs embodied in these approaches to foster the general development of U15 attackers. Although the U17 have also shown some improvements, such as the increase the CMJ, the number of successful shots as well as the individual space explored, higher effects were found in the U15 age group. Therefore, it is possible that players of older age groups, such as the U17 age group, may require training programs of higher duration and more fluctuations due the high amount of time spent in tasks with rigid tactical behavior. Overall, coaches could use training programs sustained in differential learning to improve in-game performance of youth football players.
